# Supplementary material for: Colon Bioaccessibility under In Vitro Gastrointestinal Digestion of a Red Cabbage Extract Chemically Profiled through UHPLC-Q-Orbitrap HRMS
Source: Antioxidants (Basel). 2020 Oct 6;9(10):955. doi: 10.3390/antiox9100955 (PMC7601900; doi:10.3390/antiox9100955)
Supplement: Supplementary file 1 [file antioxidants-09-00955-s001.pdf]

Table S1. Chromatographic and spectrometric parameters including retention time, adduct ion, theoretical and measured mass (*m*/*z*), accuracy and sensibility for anthocyanins (n=20) in the investigated red cabbage extract. RT = Retention Time

Tentative assignment	RT (min)	Chemical Formula	$[M]^+$ or $[M+H]^+$ theoretical mass (*m*/*z*)	$[M]^+$ or $[M+H]^+$ found Mass (*m*/*z*)	MS/MS fragment ions (*m*/*z*)	Accuracy (Δ ppm)	LOD (mg/kg)	LOQ (mg/kg)
Cyanidin 3-diglucoside 5-glucoside	3.07	$C_{33}H_{41}O_{21}$	773.21348	773.21057	611.06012- 449.16882-287.05219	-3.763	-	-
Cyanidin 3-soph 5-xyloside	3.17	$C_{32}H_{39}O_{20}$	743.20292	743.20294	611.06012- 287.97883	0.026	-	-
Cyanidin 3,5-diglucoside	3.18	$C_{27}H_{31}O_{16}$	611.16066	611.16022	449.19708-287.06469	-0.719	0.013	0.039
Cyanidin 3-galactoside	3.23	$C_{21}H_{21}O_{11}$	449.10784	449.10654	287.05576	-2.894	0.026	0.078
Cyanidin 3-(sin)soph-5-glucoside	3.25	$C_{44}H_{51}O_{25}$	979.27139	979.27136	817.19598-447.61975-287.05479	-0.030	-	-
Cyanidin 3-(sin) triglucoside-5-glucoside	3.32	$C_{50}H_{61}O_{24}$	1141.32422	1141.32544	979.27155-817.21722-449.10831-287.05466	1.068	-	-
Cyanidin 3-(glucofer)-diglucoside-5-glucoside	3.34	$C_{49}H_{59}O_{29}$	1111.29252	1111.29602	949.26074-703.49890-449.10782-287.05457-177.00717	3.419	-	-
Cyanidin 3-(*p*-coum)glucoside-5-glucoside	3.39	$C_{42}H_{57}O_{27}$	757.19744	757.19641	449.10791-287.14624	-1.360	-	-
Cyanidin 3-(caf)diglu-5-glucoside	3.47	$C_{42}H_{47}O_{24}$	935.24518	935.24414	773.21832-449.10736-287.05432	-1.112	-	-
Cyanidin 3-(fer)glucoside-5-glucoside	3.52	$C_{37}H_{39}O_{19}$	787.20800	787.20679	449.10709-287.14661	-1.537	-	-
Cyanidin 3-(sin)glucoside-5-glucoside	3.54	$C_{38}H_{41}O_{20}$	817.21857	817.21796	449.10709-287.10703	-0.746	-	-
Cyanidin 3-(*p*-coum)diglucoside-5-glucoside	3.54	$C_{42}H_{47}O_{23}$	919.25026	919.24945	757.19755-287.05457-147.06674	-0.881	-	-
Cyanidin 3-(fer)soph-5-glucoside	3.56	$C_{43}H_{49}O_{24}$	949.26083	949.26025	747.42340-449.20041-287.05457-177.00108	-0.881	-	-
Cyanidin	3.57	$C_{15}H_{11}O_6$	287.05501	287.05472	207.05879-147.07649	-0.611	0.013	0.039
Cyanidin 3-(sin)-diglucoside-5-glucoside	3.58	$C_{44}H_{51}O_{25}$	979.27139	979.27106	817.21771-447.22711-207.06528	-0.336	-	-
Cyanidin 3-(caf)(*p*-coum)diglucoside-5-glucoside	3.60	$C_{51}H_{53}O_{26}$	1081.28196	1081.28271	919.24951-756.43555-287.14618-147.06679	0.693	-	-
Cyanidin 3-(caf)(sin)soph-5-glucoside	3.61	$C_{53}H_{57}O_{28}$	1141.30309	1141.30261	979.27087-817.21808-287.05457-207.06522-163.03885	-0.420	-	-
Cyanidin 3-(sin)(*p*-coum)soph-5-glucoside	3.63	$C_{53}H_{57}O_{27}$	1125.30817	1125.30872	979.27112-287.28288-207.00906-147.06674	0.488	-	-
Cyanidin 3-(sin)(fer)soph-5-glucoside	3.65	$C_{54}H_{59}O_{28}$	1155.31874	1155.31836	993.28876-949.26105-287.05457-207.06522-177.00697	-0.330	-	-
Cyanidin 3-(sin)(sin)soph-5-glucoside	3.69	$C_{55}H_{61}O_{29}$	1185.32930	1185.32959	1023.27771-449.10782-287.05499-207.06532	0.240	-	-

*sin= sinapoyl; soph= sophoroside; fer= feruloyl; *p*-coum=*p*-cumaroyl; caf=caffeoyl; glucofer=glucoferoyl.

Table S2. Chromatographic and spectrometric parameters including retention time, adduct ion, theoretical and measured mass (*m*/*z*), accuracy and sensibility for phenolic acids and flavonoids (n=20) in the investigated red cabbage extract. RT = Retention Time

Tentative assignment	RT (min)	Chemical Formula	$[M\text{-}H]^-$ theoretical mass (*m*/*z*)	$[M\text{-}H]^-$ found mass (*m*/*z*)	MS/MS fragment ions (*m*/*z*)	Accuracy (Δ ppm)	LOD (mg/kg)	LOQ (mg/kg)
Protocatechuic acid	2.42	$C_7H_6O_4$	153.01930	153.01857	109.02840	-4.77064	0.026	0.078
Epigallocatechin	2.93	$C_{15}H_{14}O_7$	305.06675	305.06650	219.06580-159.10190-121.02846-109.02807	-0.81949	0.013	0.039
Chlorogenic acid	3.00	$C_{16}H_{18}O_9$	353.08780	353.08798	191.05594-84.98998	0.50979	0.013	0.039
Epicatechin	3.17	$C_{15}H_{14}O_7$	289.07176	289.07202	221.94647-203.09201-161.04478	0.89943	0.013	0.039
Gallocatechin	3.19	$C_{15}H_{14}O_8$	305.06676	305.06681	219.06254-159.10185-109.02836-121.02847	0.16390	0.013	0.039
Caffeic acid	3.25	$C_9H_8O_4$	179.03498	179.03455	134.99960	-2.40177	0.013	0.039
Vanillic acid	3.30	$C_8H_8O_4$	167.03490	167.03428	151.03905-123.04387	-3.71180	0.026	0.078
Catechin	3.34	$C_{15}H_{14}O_6$	289.07175	289.07205	247.02241-205.10712-151.03923-125.02335	1.03780	0.026	0.078
Syringic acid	3.39	$C_9H_{10}O_5$	197.04555	197.04503	182.02153-166.99791	-2.63898	0.026	0.078
Daidzein	3.40	$C_{15}H_9O_4$	253.05063	253.04977	209.96429-225.00984	-3.39853	0.103	0.310
p-coumaric acid	3.47	$C_9H_8O_3$	163.04001	163.03937	119.04917	-3.92542	0.026	0.078
Isoquercetin	3.65	$C_{21}H_{20}O_{12}$	463.08820	463.08853	431.09848-187.09698-174.95542	0.71261	0.013	0.039
Rutin	3.65	$C_{27}H_{30}O_{16}$	609.14611	609.14673	300.99911-271.05026-255.12390	1.01782	0.013	0.039
Ellagic acid	3.74	$C_{14}H_6O_8$	300.99899	300.99911	245.91669-229.93712-185.01208-117.00336	0.39867	0.013	0.039
Quercetin	3.97	$C_{15}H_{10}O_7$	301.03538	301.03508	174.95551	-0.99656	0.013	0.039
Naringenin	4.10	$C_{15}H_{12}O_5$	271.06120	271.06110	235.92595-151.03917	-0.36892	0.013	0.039
Kaempferol	4.13	$C_{15}H_{10}O_6$	285.04046	285.04086	93.00679	1.40331	0.013	0.039
Genistein	4.18	$C_{15}H_{10}O_5$	269.04554	269.04562	241.14435-213.14908-151.03935	0.29735	0.013	0.039
Ferulic acid	4.84	$C_{10}H_{10}O_4$	193.05063	193.05016	178.02666-149.06009-134.99963	-2.43459	0.013	0.039
Sinapic acid	5.07	$C_{11}H_{12}O_5$	223.06120	223.06100	208.93591-179.03423	-0.89661	0.026	0.078
